# Supplementary material for: The Transcriptional Landscape of Immune-Response 3′-UTR Alternative Polyadenylation in Melanoma
Source: Int J Mol Sci. 2024 Mar 6;25(5):3041. doi: 10.3390/ijms25053041 (PMC10931711; doi:10.3390/ijms25053041)
Supplement: Supplementary file 1 [file ijms-25-03041-s001.zip › Supplementary Figures.pdf]

The Transcriptional Landscape of Immune-Response 3'-UTR Alternative Polyadenylation in Melanoma

Xiao Yang, Yingyi Wu, Xingyu Chen, Jiayue Qiu and Chen Huang *

Dr. Nesher's Biophysics Laboratory for Innovative Drug Discovery, State Key Laboratory of Quality Research in Chinese Medicine, Macau University of Science and Technology, Taipa, Macao SAR 999078, China

* Authors to whom correspondence should be addressed

Supplementary Figures

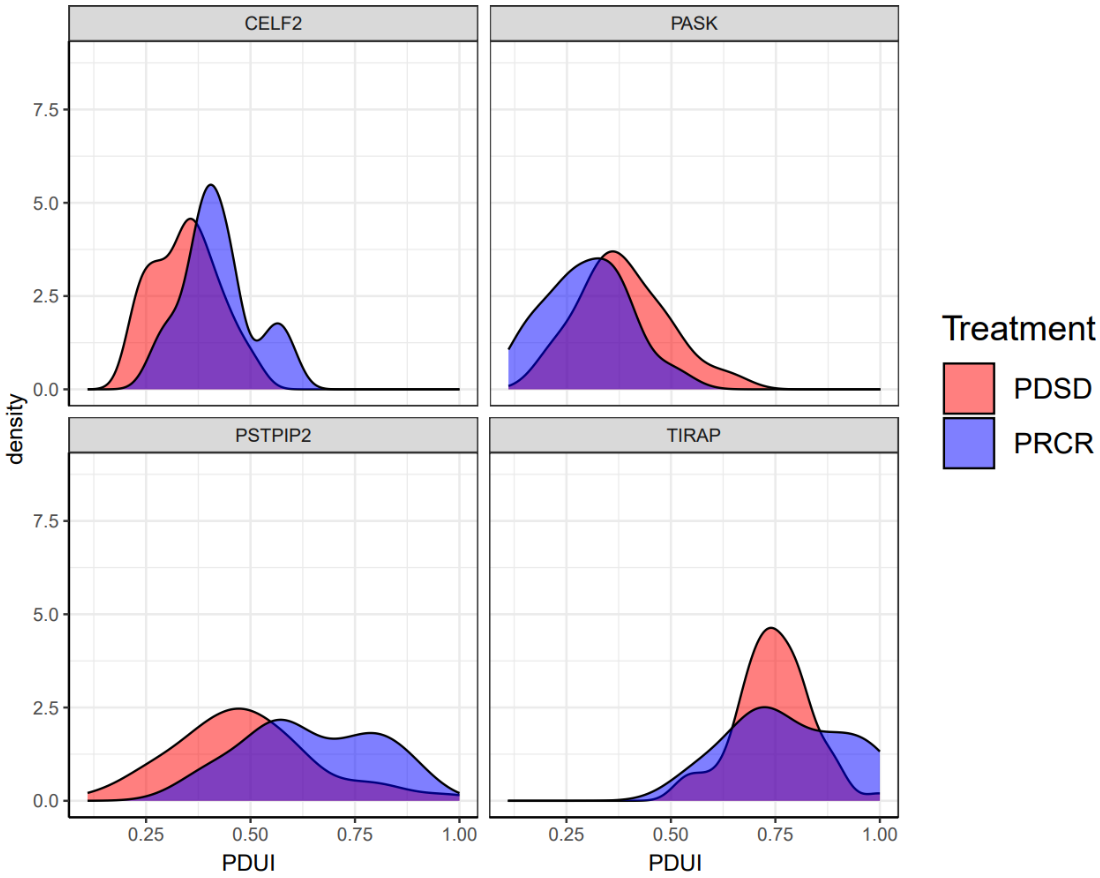

Figure S1. Differences in PDUI scores between groups of parental genes for APA events co-regulated by multiple master APA factors.

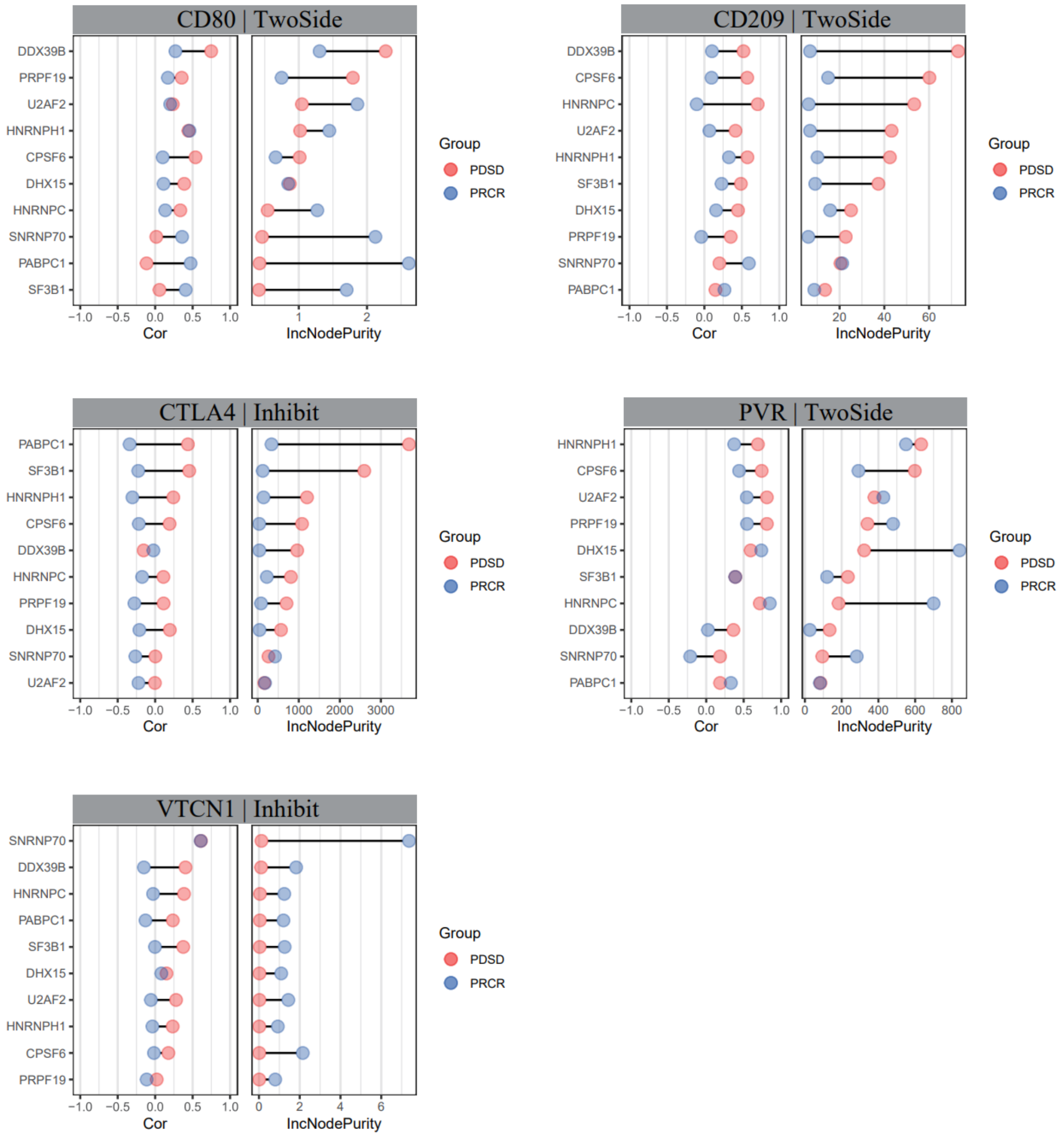

Figure S2. The correlation between the expression of multiple immune checkpoints and the expression of master APA factors, as well as the differences in the degree of correlation between groups, were analysed in the GSE78220 patient cohort.

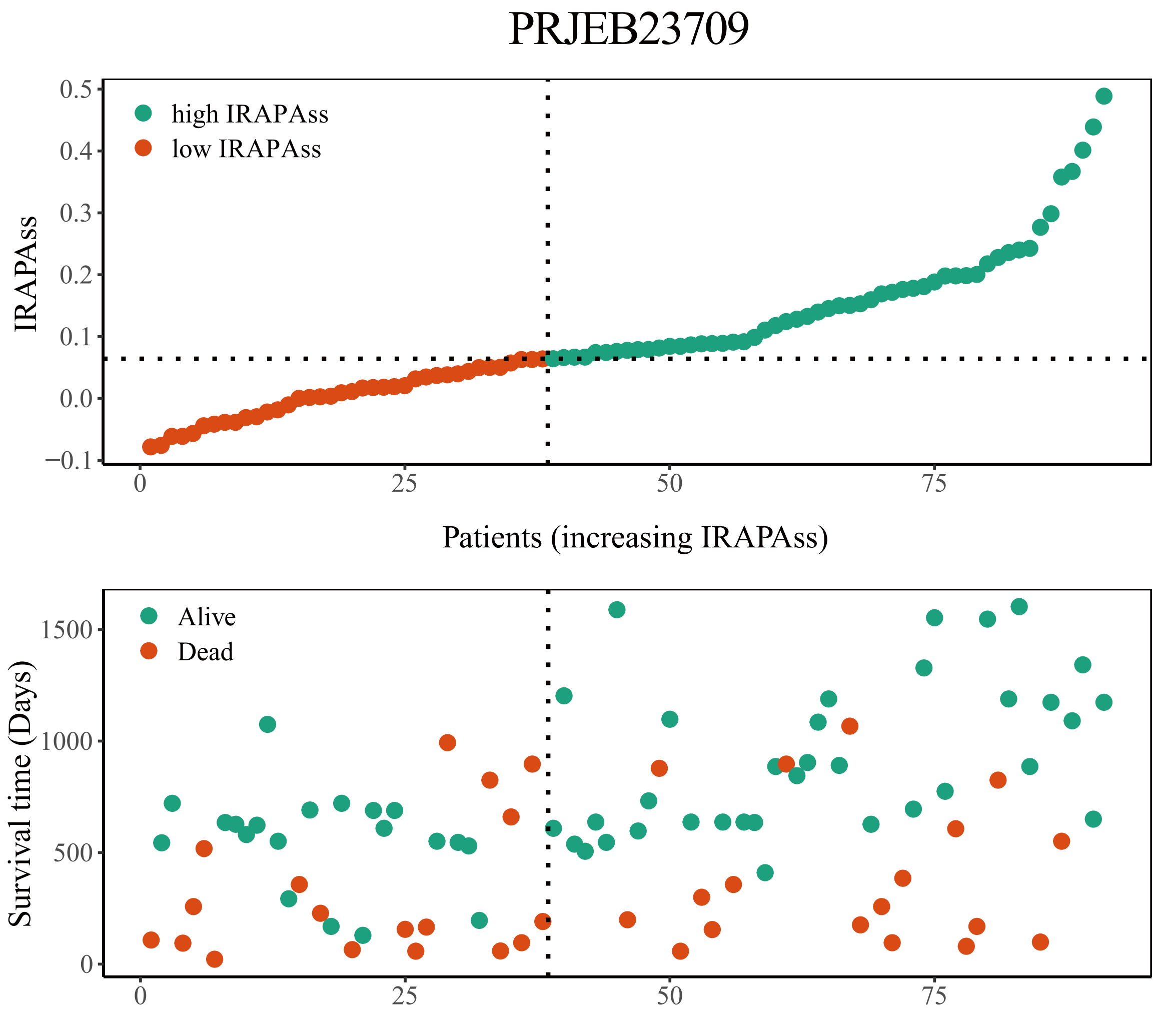

Figure S3. Dot plot: According to the optimal cutoff value, the samples were divided into a high IRAPAss and a low IRAPAss group.

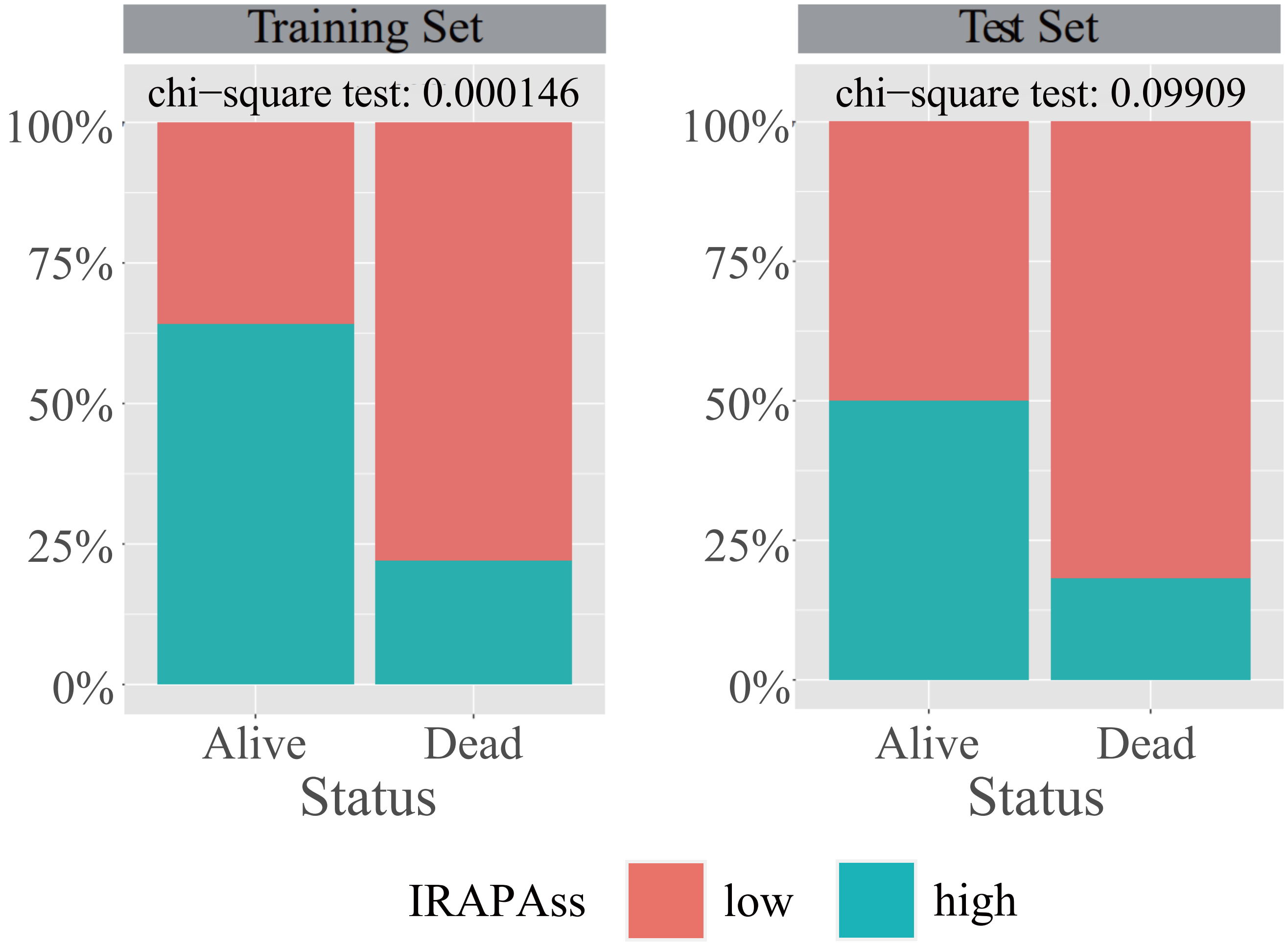

Figure S4. Histogram of the distribution of high and low IRAPAss between responders and non-responders in the training data and testing data

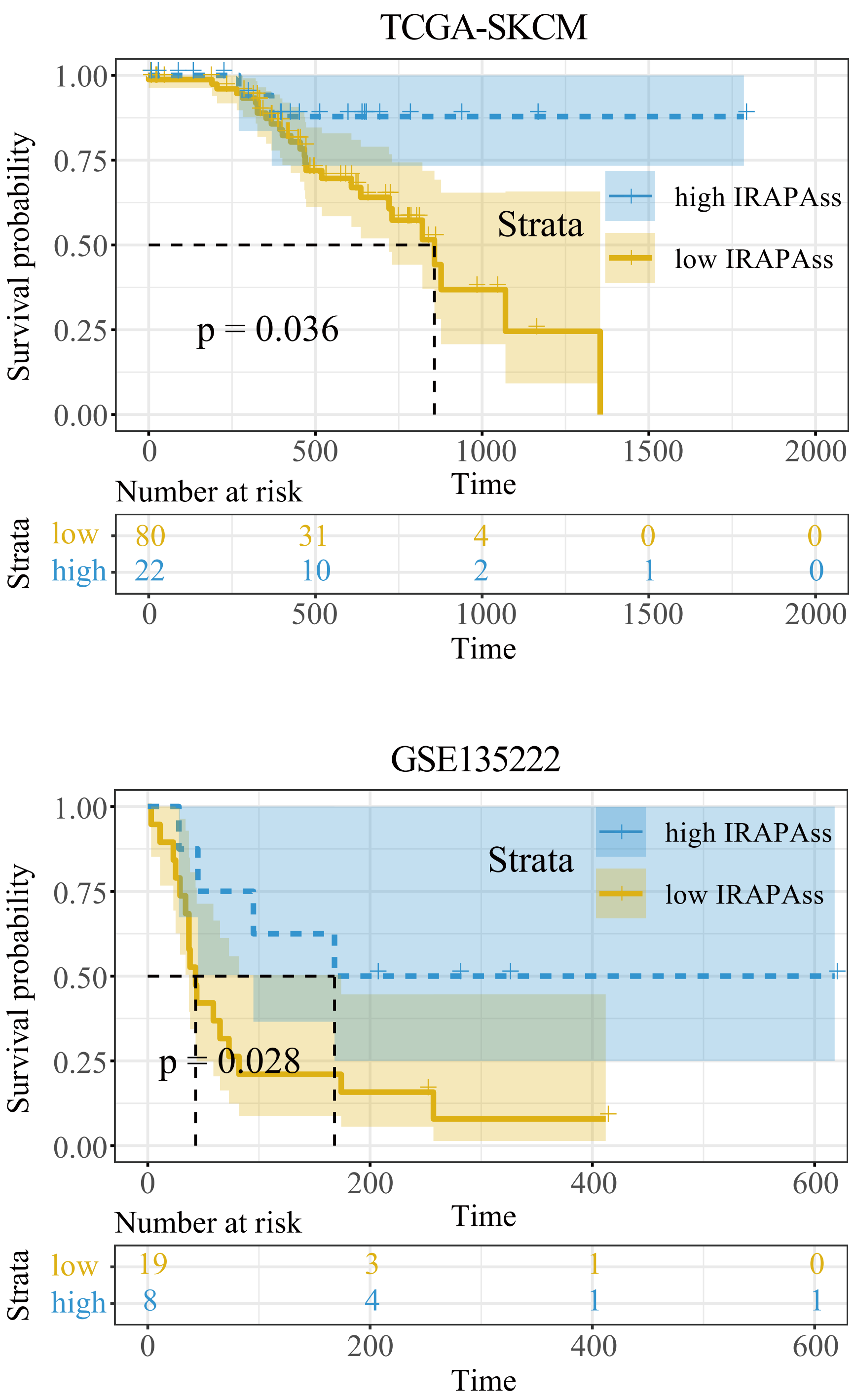

Figure S5. Kaplan-Meier curves for the TCGA-SKCM and GSE135222 cohorts.

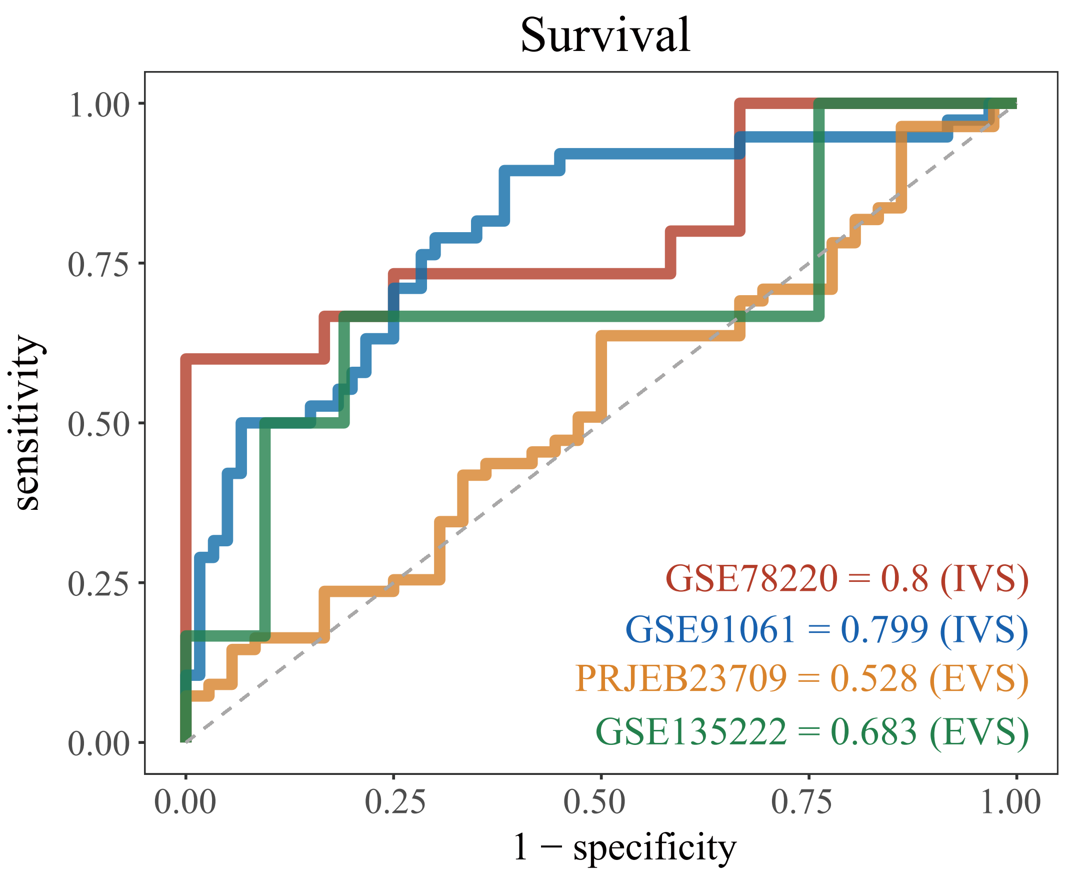

Figure S6. The ROC curves of Survival signatures in four cohorts.

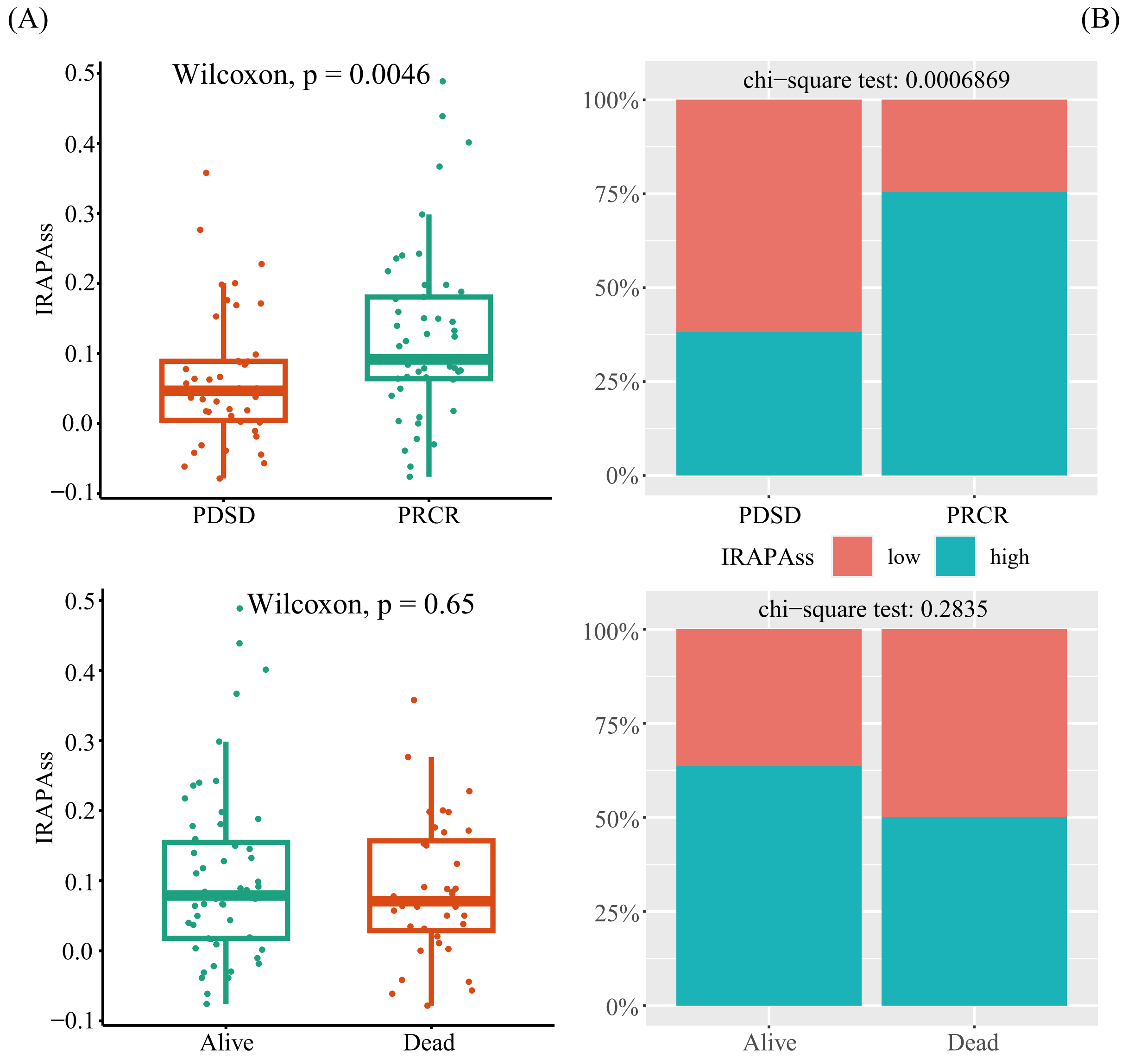

Figure S7. (A) Boxplots of IRAPAss distributions for responders and non-responders, surviving and deceased patients in the PRJEB23709 cohort. (B) Histogram of the distribution of high and low IRAPAss for responders and non-responders, surviving and decreased patients in the PRJEB23709 cohort.

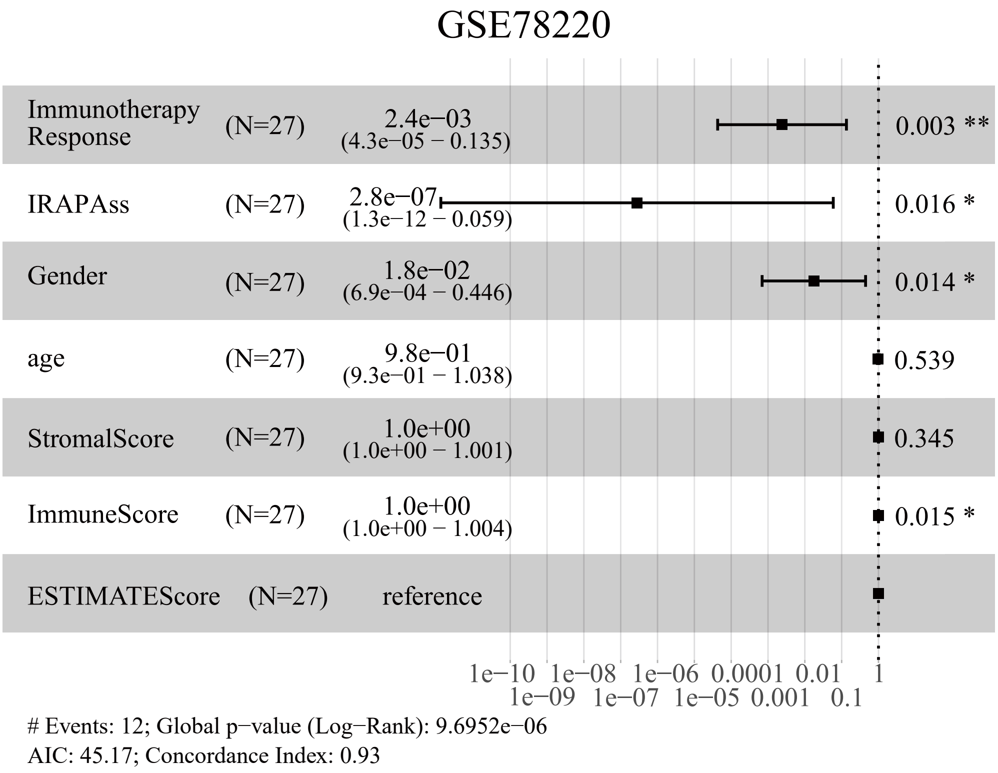

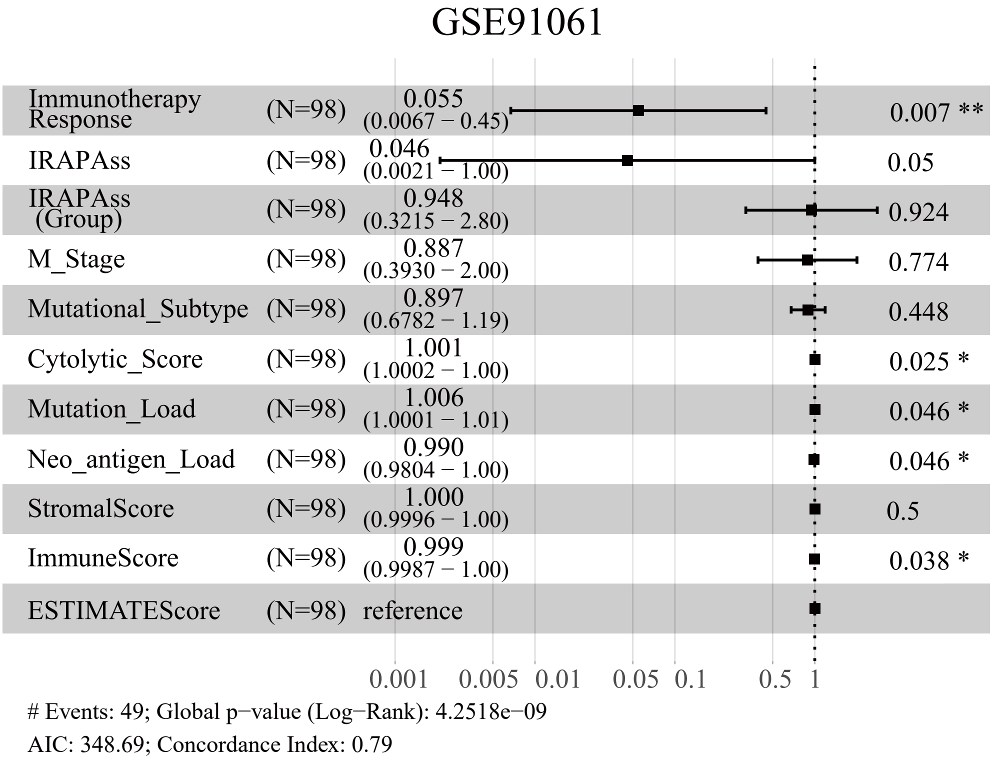

Figure S8. multivariate Cox regression analysis of IRAPAss and melanoma clinical data in the GSE78220 and GSE91061 cohorts. The P values of the figures are shown as follows: *, $P < 0.05$. **, $P < 0.01$.

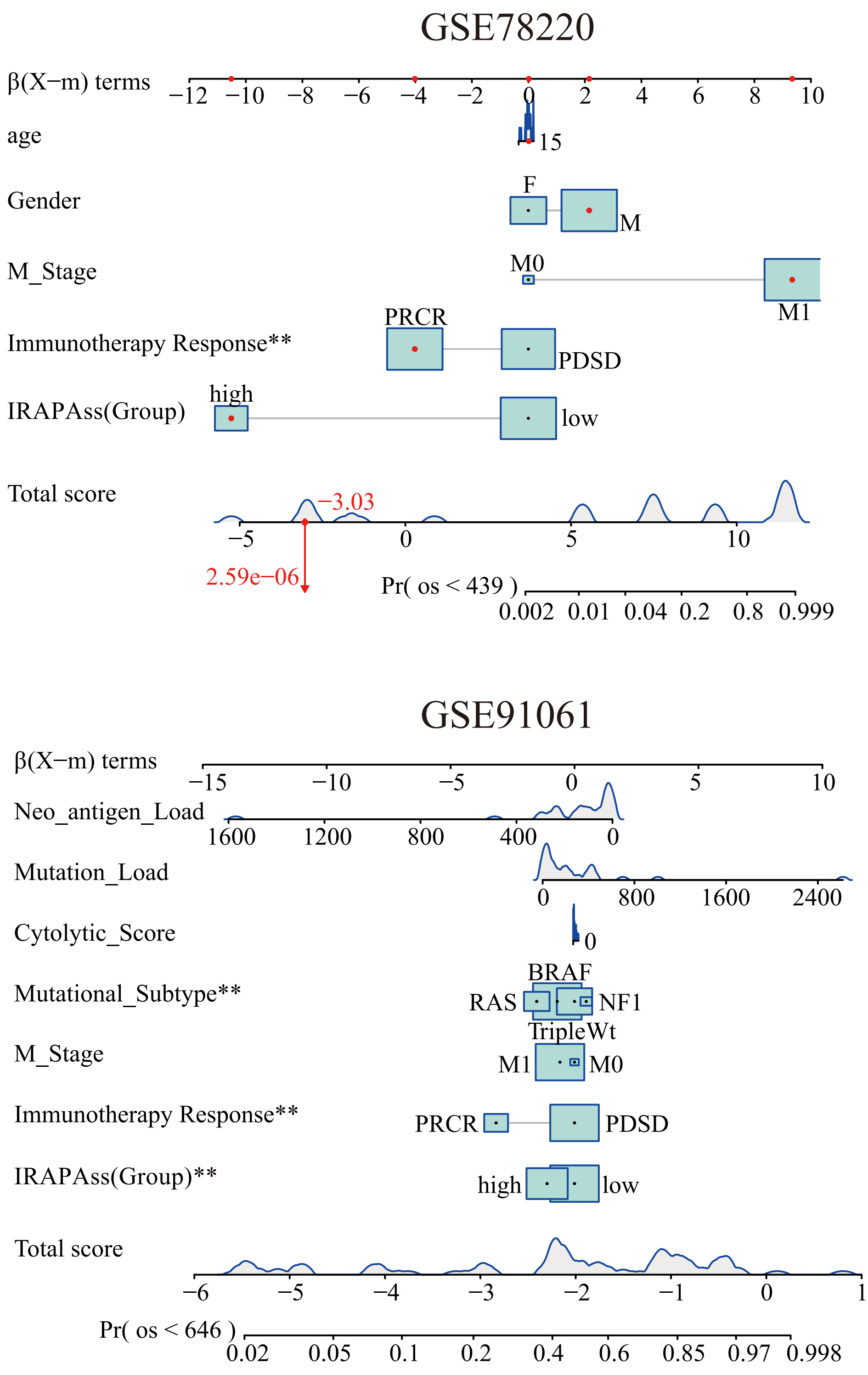

Figure S9. Nomograms containing IRAPAss and prognostic clinical data were built to predict the OS of melanoma patients in the GSE78220 and GSE91061 cohorts. The P values of the figures are shown as follows: **, P < 0.01.

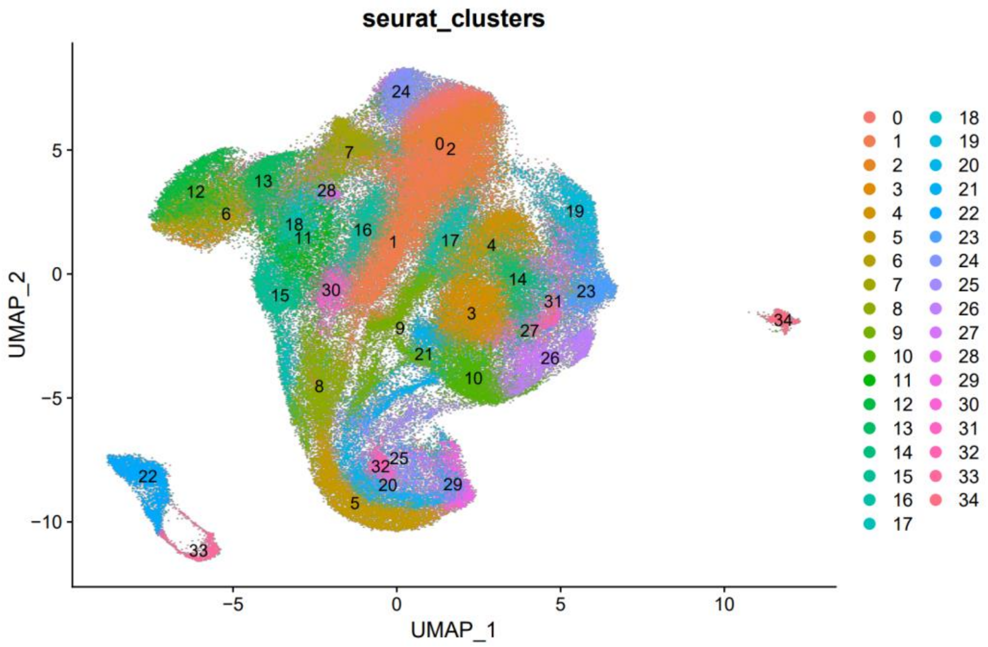

Figure S10. Unsupervised clustering of 186227 T cells by UMAP.

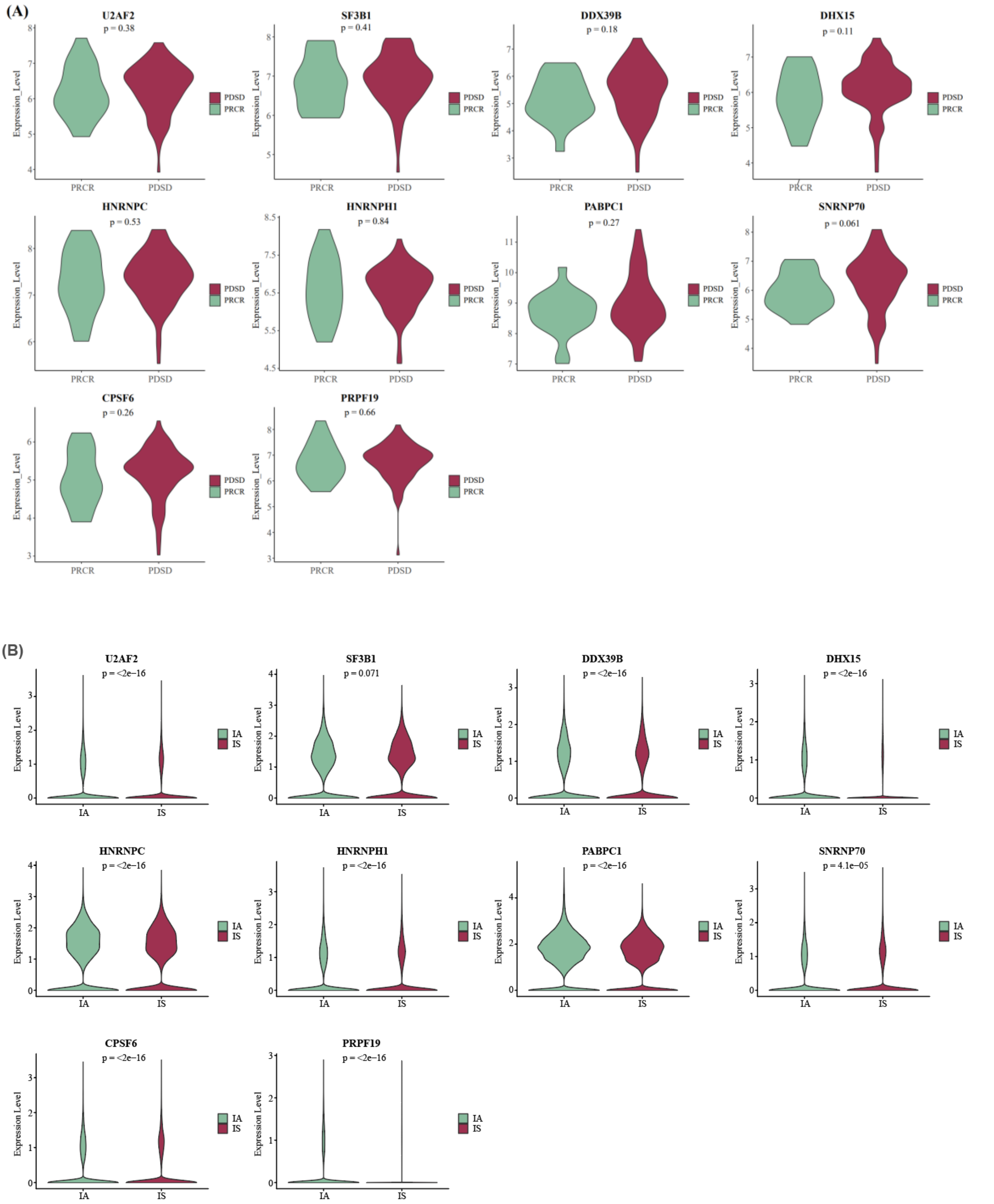

Figure S11. (A) Differences in the Expression of master APA factors between responders and non-responders at the Bulk RNA-seq level. (B) Differences in the Expression of master APA factors between immunoactivation (IA) and immunosuppression (IS) T cells at the scRNA-seq level.

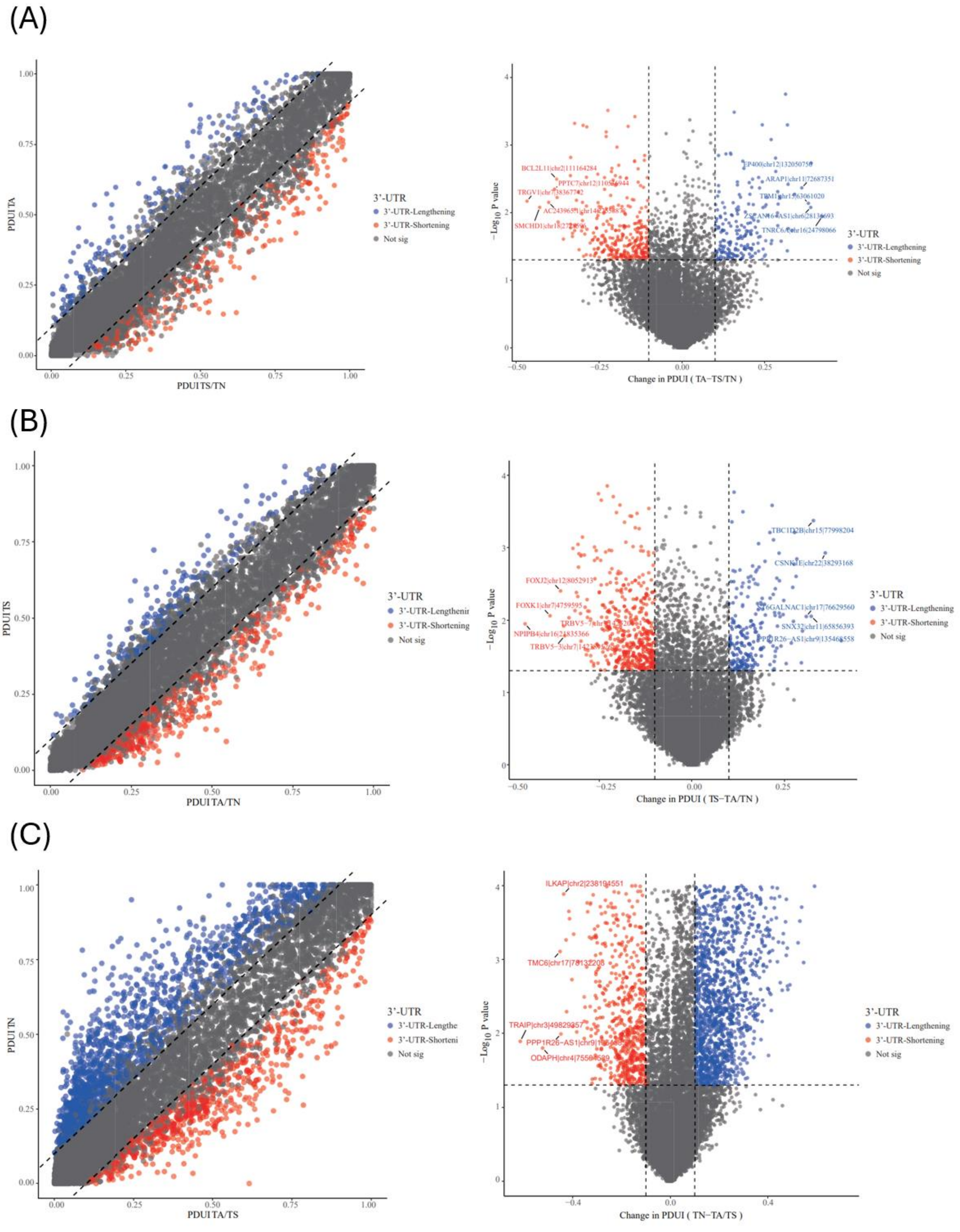

Figure S12. The results of the analysis of the differences between the groups for each APA event are presented in the form of mean PDUI score curves and volcano plots. (A) TA vs TS + TN. (B) TS vs TA + TN. (C) TN vs TA + TS.

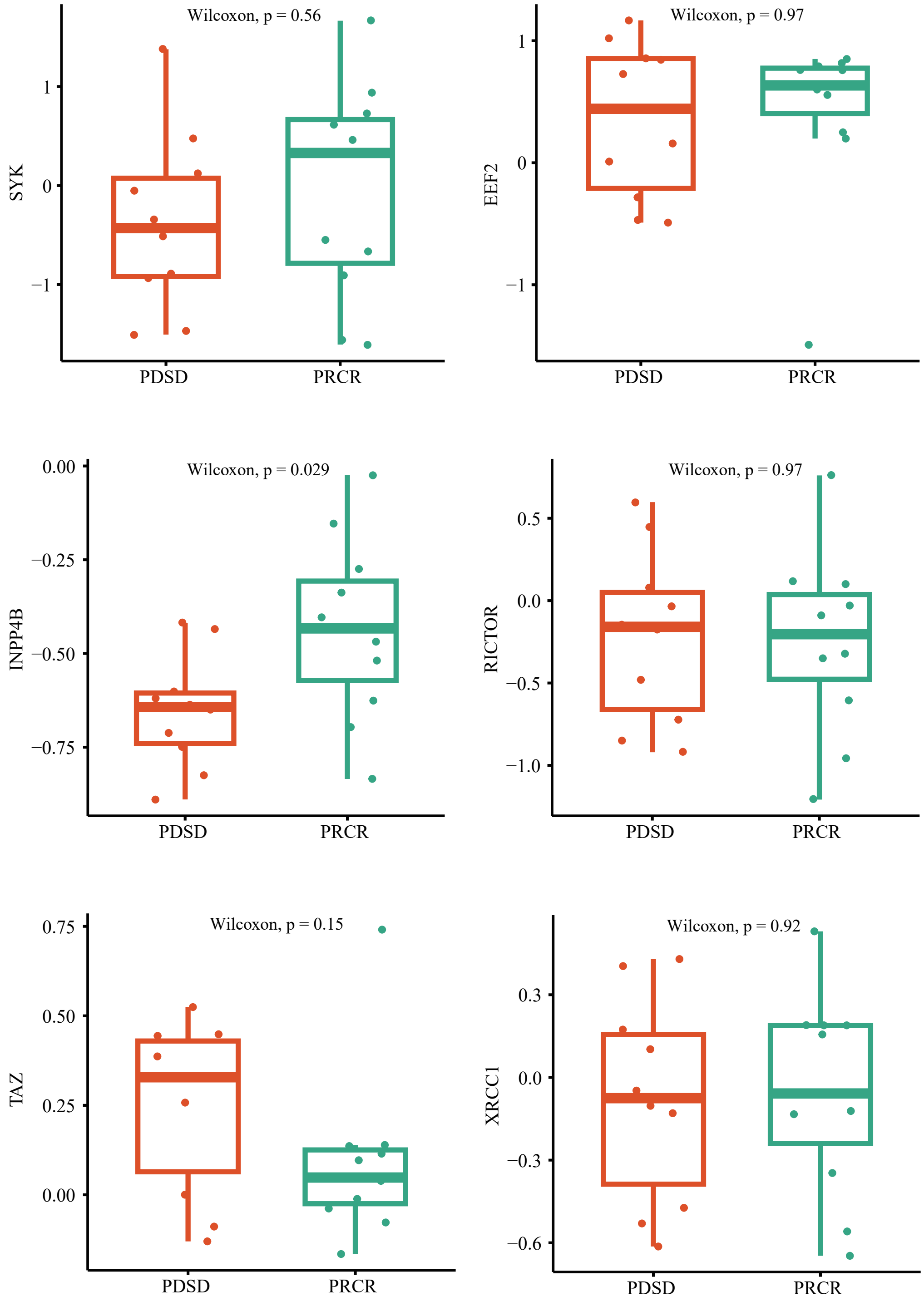

Figure S13. The signal intensity distribution of protein expression in responders and non-responders in the TCGA-SKCM cohort.

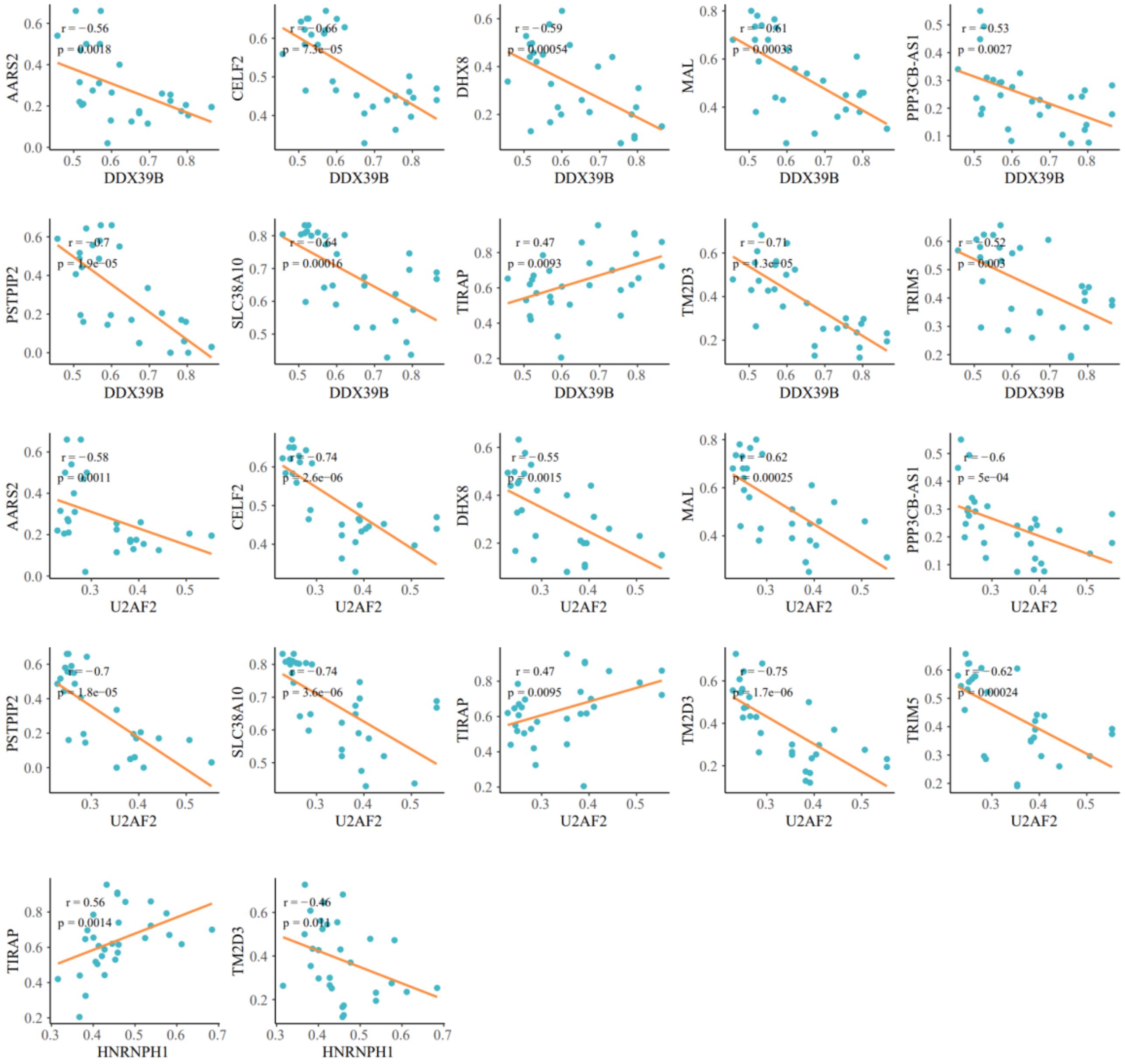

Figure S14. The dot plots illustrate the correlation trends between master APA factors and APA event parental genes that are identical to the subnetwork
